# Impact of Serum Phosphate on Hemoglobin Level: A Longitudinal Analysis on a Large Cohort of Dialysis Patients

**DOI:** 10.3390/jcm13195657

**Published:** 2024-09-24

**Authors:** Vincenzo Calabrese, Giovanni Luigi Tripepi, Domenico Santoro, Valeria Cernaro, Vincenzo Antonio Panuccio, Sabrina Mezzatesta, Francesco Mattace-Raso, Claudia Torino, on behalf of the Sicilian Registry of Nephrology, Dialysis and Transplantation

**Affiliations:** 1 Department of Internal Medicine, Erasmus MC, University Medical Center Rotterdam, 3015 GD Rotterdam, The Netherlands; v.calabrese@outlook.it (V.C.); claudia.torino@cnr.it (C.T.); 2 Department of Medicine and Surgery, University of Enna “Kore”, 94100 Enna, Italy; 3 National Research Council—Institute of Clinical Physiology, 89124 Reggio Calabria, Italy; gtripepi@ifc.cnr.it (G.L.T.); sabrinamezzatesta@cnr.it (S.M.); 4 Unit of Nephrology and Dialysis, Department of Clinical and Experimental Medicine, A.O.U. “G. Martino”, University of Messina, 98122 Messina, Italy; dsantoro@unime.it (D.S.); vcernaro@unime.it (V.C.); 5 GOM “Bianchi Melacrino Morelli”, Nephrology Unit, 89124 Reggio Calabria, Italy; enzopanuccio@gmail.com

**Keywords:** dialysis, hemoglobin, FGF23, serum phosphate, longitudinal analysis

## Abstract

**Background/Objectives**: Phosphate is a macro-element involved in all cellular energetic processes. As about 90% of the phosphate filtered by the glomerulus is excreted by kidneys, the impairment of renal function and the consequent over-secretion of parathyroid hormone and fibroblast growth factor 23 results in the increase in the serum phosphate levels. The association between phosphate and hemoglobin is controversial, as both direct and indirect relationships have been reported. The present study aims to investigate the relationship between phosphate and hemoglobin in a large prospective, longitudinal cohort including dialysis patients from the Sicilian Registry of Nephrology, Dialysis, and Transplantation. **Methods**: In this prospective cohort study, we included 6263 hemodialysis patients to achieve a total of 120,462 repeated measurements of serum phosphate and hemoglobin over time. The longitudinal association between phosphate and hemoglobin was analyzed by univariate and multivariate Linear Mixed Models. **Results:** The mean age was 66 ± 16 years and the median dialysis vintage was 5 months [IQR: 2–16]. Mean and median values of hemoglobin and phosphate were 10.7 g/dL (SD 1.3 g/dL) and 4.6 mg/dL [IQR 3.9–5.5 mg/dL], respectively. The multivariate model, adjusted for potential confounders, confirmed the positive association between serum phosphate and hemoglobin [adjβ = 0.13, 95%CI 0.03–0.23, *p* = 0.01)]. These results were confirmed in analyses stratified for the use of phosphate binders. **Conclusions:** In our large cohort of dialysis patients, we found a linear, direct relationship between phosphate and hemoglobin levels. As a reduction in phosphate is associated with a parallel reduction in hemoglobin levels, hypophosphatemia can accentuate anemia in dialysis patients. Our results generate the hypothesis that monitoring serum phosphate in clinical practice might provide a better management of anemia.

## 1. Introduction

Phosphate is a macro-element involved in all cellular energetic processes. Approximately 85% is deposited as crystals of hydroxyapatite in the bone, whereas the remaining 15% is stored in intracellular space as a component of nucleic acids and extracellular matrix [1]. About 90% of the phosphate filtered by the glomerulus is excreted by kidneys; thus, the reduction in nephron mass and the impairment of renal function are responsible for a reduction in phosphate excretion. As a consequence of this, from the early stages of chronic kidney disease (CKD) [2], parathyroid hormone (PTH) and fibroblast growth factor 23 (FGF-23), two hormones with phosphaturic activity, are over-secreted with the aim to regulate the serum concentration of this ion. Considering that dialysis patients are anuric, in this population, the over-secretion of the two hormones results in hyperphosphatemia, which, via FGF23, results in iron deficiency and anemia [3].

Although, in CKD patients, anemia is mainly caused by reduced erythropoietin levels [4,5,6], its onset has a multifactorial etiology. As proof of this, not only hyperphosphatemia but also hypophosphatemia impairs the hemoglobin level, suggesting a U-shaped association between phosphate and hemoglobin. Hypophosphatemia has been related to hemoglobin reduction both in experimental studies and in case reports. Low phosphate levels impair red blood cells’ structure, with consequent hemolysis [7,8,9,10]. In addition, it reduces the stability of erythrocytes’ membranes [11] and biconcavity [12] and increases hemoglobin oxygen affinity, which is in turn negatively related to hemoglobin concentration [13,14] Furthermore, hypophosphatemia increases phosphofructokinase activity and reduces ATP and the erythrocyte concentrations of glucose-6-phosphate and fructose-6-phosphate [15,16,17,18]. 

Due to the low prevalence of hypophosphatemia, current evidence mostly comes from case reports or case series, with only a small observational study (including 23 patients) and a case report involving uremic patients confirming this hypothesis [19,20]. Conversely, the impact of high serum phosphate on hemoglobin is much more studied, even though longitudinal studies are not available yet. 

To our knowledge, no studies investigating the association between phosphate and hemoglobin and performed exclusively on dialysis patients have been published so far. More specifically, the association between serum phosphate and hemoglobin has never been longitudinally evaluated in dialysis patients. 

The present study aims to investigate the possible association between repeated measurements of serum phosphate and hemoglobin levels in a large prospective cohort including dialysis patients from the Sicilian Registry of Nephrology, Dialysis, and Transplantation.

## 2. Materials and Methods

The study is in conformity with the guidelines of the Italian Data Protection Authority and in agreement with the Helsinki declaration. Ethical approval was not necessary based on the fact that the Sicilian Registry of Nephrology, Dialysis, and Transplantation [21] is a collection of regional data, instituted by regional laws. The Sicilian registry was created in 2008, with a decree (03423/08) that established its aim, i.e., collecting data and analyzing it. Informed consent is requested from all patients whose data are entered in the registry. However, as specified in art. 1, no formal approval from ethical committees is needed to analyze data as they are made available only in anonymous form.

### 2.1. Study Population Ad Laboratory Data

In this prospective cohort study, we included hemodialysis patients entered in the Sicilian Registry of Nephrology, Dialysis, and Transplantation from 1 January 2018 to 31 December 2020. Hemoglobin and phosphate measurements were collected from the start of the dialysis treatment. The median length of follow up was 52 [22–93] months. During this time, patients had a median number of 16 [6–39] measurements of hemoglobin and potassium. 

**Figure 1:**
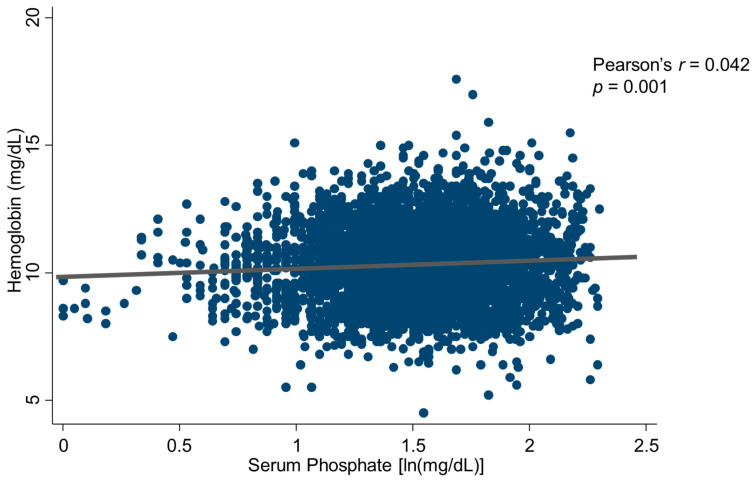
Scatterplot of the inter-relationship between serum phosphate and hemoglobin.

Of the 6451 patients composing the original cohort, 101 were excluded because they were affected by hematologic disorders, and 87 were excluded because of missing serum phosphate and/or hemoglobin measurements, leaving 6263 hemodialysis patients, which facilitated a total of 120,462 repeated measurements of serum phosphate and hemoglobin over time (Figure 1). 

Patients had been on regular hemodialysis for a median time of 36 months (inter-quartile range: 10–78) and were being treated with standard bicarbonate dialysis with non-cellulosic membrane filters of various types (Enaxone, Elixone, Polyethersulfon, Polyacrylonitrile, Polyamide, Polymix, Polyethylene–Polyvin–Alcohol, Polymethylmethacrylate). In total, 3018 patients were treated with various anti-hypertensive drugs (874 on mono-therapy with ACE inhibitors, calcium channel blockers, α- and β-blockers, vasodilators, diuretics, or other drugs; 1033 on double therapy; 728 on triple therapy; and 383 patients on quadruple or quintuple therapy with various combinations of these drugs). The main demographic, somatometric, clinical, and biochemical characteristics of the study population are detailed in Table 1A,B.

### 2.2. Data Collection

Laboratory and clinical data were collected locally from the Register referents as part of the normal clinical practice and then entered in the platform. Laboratory data included serum phosphate, hemoglobin, C-reactive protein, iron, transferrin, ferritin, potassium, calcium, intact PTH, albumin, glucose, triglycerides, cholesterol, bicarbonate, alkaline phosphatase, fractional urea clearance (Kt/V), and β2-microglobulin. Clinical data included blood pressure levels, residual diuresis, previous comorbidities (dementia, hemiplegia, liver disease, history of arterial hypertension, vascular disease, chronic obstructive pulmonary disease (COPD) malignancy with/without metastasis, heart failure, psychiatric disease, dyslipidemia, prostatic hypertrophy) as well as pharmacological treatment such as anti-hypertensives, folic acid, calcium carbonate, cholecalciferol, insulin, aspirin, allopurinol, phosphorous binder, calcium-mimetics, cortisone, erythropoiesis-stimulating factors (ESA), iron supplementation, immunosuppressive treatment, proton pump inhibitors, paricalcitol, and vitamin B12. Details of the registry are described elsewhere [21].

### 2.3. Statistical Analysis 

Data were described as mean ± standard deviation, median and interquartile range, or proportion, as appropriate. Hemoglobin and phosphate, as well as the quantitative confounders, were included in the analysis as continuous variables. The distribution of variables was investigated by the Kolmogorov–Smirnov test followed by graphic evaluation. The number of missing data varied across variables. In detail, albumin was missing in about of 50% of measures, potassium in 38%, calcium in 22%, BMI in 21%, PTH in 58%, ferritin in 58%, CRP 75%, HCO_3_ 74%, and systolic blood pressure in 12%. Other variables included in the multivariate models had less than 0.01% missing data. Missing values were related neither to the center which provided the data nor to specific characteristics of the patients, so were considered at random. As mixed effects models are well equipped to handle missing (at random) response data if estimated using likelihood methods, we did not impute or recover them. 

Baseline hemoglobin was described in the whole sample and according to quartiles of serum phosphate and compared using ANOVA test and Bonferroni post hoc analysis. The first quartile included patients with serum phosphate lower than 3.9 mg/dL (*n* = 1597), the second quartile, patients with serum phosphate between 3.91 and 4.5 mg/dL (*n* = 1456), the third quartile, patients with serum phosphate between 4.51 and 5.5 mg/dL (*n* = 1522), and the fourth quartile, those with serum phosphate higher than 5.51 mg/dL (*n* = 1688). The baseline correlates of serum phosphate and hemoglobin were tested by Pearson’s product moment correlation coefficient (Supplementary Table S1). The analysis was also performed after excluding potential outliers, detected by graphical evaluation and by the Grubbs method (double sides).

The longitudinal association between phosphate and hemoglobin was analyzed by univariate and multivariate Linear Mixed Models. In adjusted analyses, we included as potential confounders all variables related to serum phosphate and hemoglobin with *p* value < 0.2. As Pearson’s r was neither constant, nor constantly modified, nor contiguously modified visit to visit, the unstructured matrix was applied in the analysis. Multivariate models adjusted for sex, serum potassium, calcium carbonate, folic acid, iron supplementation, erythropoietin-stimulating agents (ESAs), paricalcitol, immunosuppressors, calcitriol, heart failure, peripheral vascular disease, diabetes, chronic liver disease, visit, KT/V, BMI, iPTH, transferrin saturation, serum ferritin, C-reactive protein (CRP), serum bicarbonate, serum calcium, systolic blood pressure (SBP), age, phosphate binders, ACE inhibitors (AceI), proton pump inhibitors (PPIs), arterial hypertension history, vitamin B12, diuretics use, cinacalcet use, dementia, malignancies without presence of metastasis, and chronic obstructive pulmonary disease (COPD) were evaluated through the analysis of residuals; the Hosman sensitivity analysis was also performed.

A sensitivity analysis was performed, computing missing values by multiple imputation using random forest methods with five sets of decisions, three for each imputation step. Final linear mixed model was also computed in this imputed database.

In order to exclude confounding by indication, the same analyses were performed when dividing the study population according to the use of phosphate binders.

To investigate the longitudinal effect of modification by age on the link between serum phosphate and hemoglobin, we included the following in the Linear Mixed Models: serum phosphate, age, and their product (serum phosphate*age).

## 3. Results

### 3.1. Baseline Analysis

The clinical, demographic, and somatometric characteristics of the whole population are shown in Table 1A,B. The mean age was 66 ± 16 years, and the median dialysis vintage was 5 months [IQR: 2–16]. Hemoglobin had a normal distribution with a mean value of 10.7 g/dL (SD 1.3 g/dL). Serum phosphate ranged from 0.89 to 9.99 mg/dL, was not normally distributed, and its median value was 4.6 mg/dL [IQR 3.9–5.5 mg/dL]. At baseline, the serum phosphate concentration was normal (i.e., 3.5–4.5 mg/dL) in 1771 patients (28.28%), low (<3.5 mg/dL) in 1003 patients (16.01%), and high (>4.5 mg/dL) in 3489 patients (55.71%). 

These splits based on phosphate quartiles did not suggest a U-shape relationship (10.3 ± 1.3 vs.10.4 ± 1.2 vs. 10.5 ± 1.3 vs. 10.4 ± 1.5); in addition, Bonferroni post hoc analysis showed significant differences between the first quartile and the others (p12 = 0.08, p13 = 0.001, p14 = 0.01), but other differences were not significant. Serum phosphate and hemoglobin levels were positively associated (r = 0.042, *p* = 0.001) (Figure 1), and both variables were related to albumin, serum calcium, dementia, diabetes, serum bicarbonate, kT/V, COPD, malignancy, phosphate binders, serum potassium, reactive c protein, and heart failure (all *p* < 0.05, Supplementary Table S1). No association was found between hemoglobin and age (r = −0.015, *p* = 0.247). A sensitivity analysis, performed excluding outliers in ferritin, confirmed the result. 

### 3.2. Longitudinal Analysis

The univariate Linear Mixed Model for repeated measures showed a direct association between serum phosphate and hemoglobin [β = 0.39, 95%CI 0.36–0.41, *p* < 0.001]. These results show that for each unitary increase in log-transformed serum phosphate, there was an augmentation of 0.39 g/dL on hemoglobin values or, in other words, for each unitary reduction in log-transformed serum phosphate, hemoglobin values were reduced by 0.39 g/dL. 

The multivariate model, adjusted for potential confounders, confirmed the positive association between serum phosphate and hemoglobin [adjβ = 0.14, 95%CI 0.04–0.24, *p* = 0.01)] (Supplementary Table S2). In more detail, ESA, serum ferritin, serum bicarbonate, and systolic pressure were inversely associated with hemoglobin, whereas the other variables included in the model showed a positive association (Supplementary Table S2). The association between phosphate and hemoglobin was confirmed in the Hosman sensitivity analysis [adjβ for each unitary increase in phosphate = 0.16, 95%CI 0.06/0.26, *p* = 0.001] (Supplementary Table S3). 

The same model computed in the imputed model did not significantly differ from the previous multivariate model, showing same direction and same significance of the association (Adjβ = 0.38, 95%CI 0.35/0.41, *p* < 0.001).

No interaction was observed between serum phosphate and number of visit.

**Figure 2:**
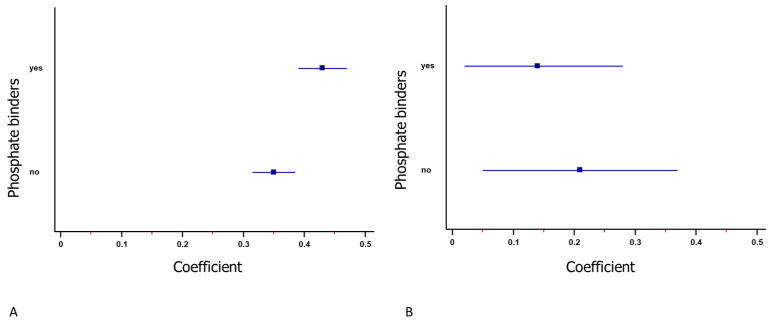
Forest plot of the association between serum phosphate and hemoglobin for patients who took phosphate binders. Both of them were positively related to hemoglobin both in univariate (**A**) and multivariate (**B**) analysis. The square represents the coefficient, and the line represents the 95% confidence interval. Upper lines show the coefficients in patients who assumed phosphorous binders, whereas lower lines show the coefficients in patients who did not assume phosphorous binders.

As EPO and phosphate binders were prescribed according to hemoglobin and serum phosphate values, we considered them as potential sources of confounding by indication. For this reason, we performed a sensitivity analysis excluding these variables. Our results showed that additional adjustment or stratification for the use of phosphate binders did not change the results (Figure 2).

**Figure 3:**
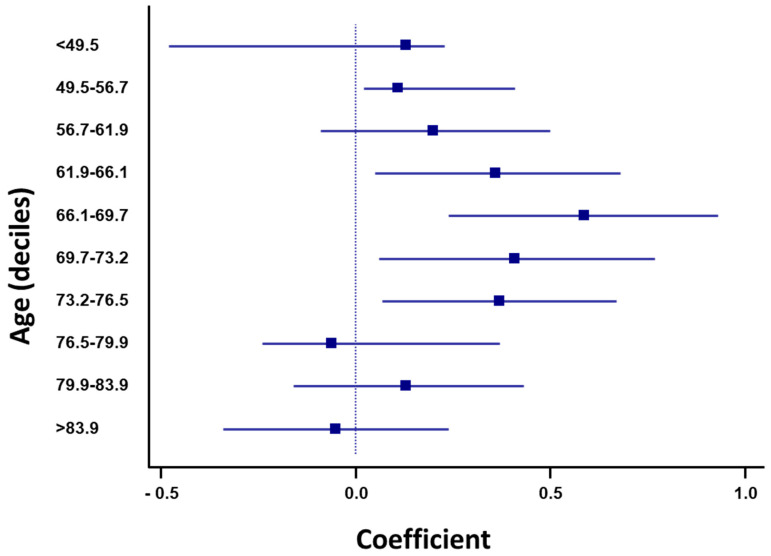
Forest plot of the association between serum phosphate and hemoglobin, stratified for deciles of age. Although no linear interaction was found, a trend was found in four deciles from 62 to 76.5 years. The square represents the coefficient, and the line represents the 95% confidence interval.

Figure 3 shows the association between phosphate and hemoglobin across age strata. In spite of the trend observed, which seems to suggest that this association was significant only in the age groups from 62 to 77 years, no effect modification by age (expressed as deciles) was observed (*p* = 0.19). 

## 4. Discussion

In the present study, we found that, in a large cohort of dialysis patients, an increase in serum phosphate was associated with high levels of hemoglobin. Such an effect is independent from comorbidities, dialysis performance, therapy, and age.

As extensively reported in the literature, hemoglobin concentration is impaired both in hypophosphatemia and hyperphosphatemia [2].

Several mechanisms are involved in hypo- or hypophosphatemia-driven anemia. Hypophosphatemia is responsible for the reduction in ATP and 2,3-diphosphoglycerate levels, which in turn decreases the stability of the erythrocyte membrane through two mechanisms: the outward inhibition of calcium and structural damage. Low erythrocyte ATP impairs the calcium pump activities and inhibits the outward calcium flow. The excess of calcium binds to spectrin, thus damaging the membrane with consequent hemolysis [9]. Furthermore, ATP plays an important role in erythrocytes’ biconcavity, so its reduction makes their structure unstable. As a direct consequence, the additional damages to the membrane and the alterations in the shape and size of erythrocytes, incompatible with the capillary flow, improves the risk of hemolysis, with consequent anemia. 

On the other hand, hyperphosphatemia impairs hemoglobin levels via the overproduction of FGF23, in turn associated with iron deficiency [22,23]. High phosphate levels induce FGF23 overproduction as a compensatory mechanism, probably in response to PTH overproduction. FGF23 overproduction induces liver overstimulation of inflammatory molecules which impair erythropoiesis, reduce EPO excretion from the kidney, and block the erythrocyte cell cycle in G2, i.e., prevent them from completing the mitosis process [4,6]. Indeed, through hepatic pro-inflammation molecule secretion, FGF23 promotes hepcidin synthesis, reducing iron availability. In addition to the FGF23-mediated pathway, high phosphate levels may directly impair the hemoglobin level through toxic products such as the fetuin A [24].

Our analysis showed a linear, direct association between phosphate and hemoglobin values, suggesting that the hypophosphatemic impact on hemoglobin (i.e., the reduction in hemoglobin due to hypophosphatemia) could overbear the hyperphosphatemic impact (i.e., the reduction in hemoglobin due to hypophosphatemia). 

Several mechanisms could explain our results. First, serum phosphate in dialysis patients is subjected to a quick reduction during the dialysis treatment, due to the low phosphate concentration in the dialysate. Thus, hyperphosphatemia could be adjusted faster than hypophosphatemia, leading to a more stable pathophysiological effect of the latter. Furthermore, the modification in the impact of phosphate on hemoglobin levels in conservative CKD could be due to the increasing FGF23, whose concentration is much higher in hemodialysis (HD) as compared to peritoneal dialysis (PD) or conservative CKD [25]. In addition, in dialysis patients, the association between FGF23 and serum phosphate seems to be less powerful than in patients in conservative treatment [26], and this may be due to the constitutively extremely high level of these hormones in this population, which may lead the body to become accustomed to them. Moreover, the impact of FGF23 on mortality is also reduced in dialysis patients [27]. Finally, serum phosphate augmentation in the interdialytic time represents a chronic process, causing tolerance and compensation mechanisms. 

Differently from phosphate, the association between iPTH and anemia has been mainly investigated only in cross-sectional studies [28]. In these studies, PTH has an impact on the hemoglobin level in patients affected by CKD in conservative treatment, but not in dialysis patients. Indeed, in several observational studies in patients affected by CKD in conservative treatment, the PTH seemed to be related to the worse management of anemia, through impairment of the erythrocytes’ median of fragility (MOF) and increased EPO resistance [29,30,31], but few studies were conducted in dialysis patients only. Among these, and in line with our results, no differences in PTH were detected [32]. 

In keeping with our hypothesis, the association between hemoglobin and age in dialysis also differed from patients in conservative treatment. In detail, whereas hemoglobin is negatively related to age in the majority of studies [33,34,35,36,37], in our cohort, no association was detected. Furthermore, in a univariate longitudinal evaluation, the relationship between hemoglobin and age seemed to be direct, even though not clinically relevant, although in conservatively treated populations, the association is often negative. This highlights the different characteristics of our cohort, which is composed only of dialysis patients, not non-dialysis patients. 

Diskin et al. [38] demonstrated that increased ESA doses are needed in patients with hyperphosphatemia. A similar hypothesis was speculated by Kamyar et al. [39] in a cohort of 49,215 patients, showing a significant positive correlation between phosphorus and EPO dose, and confirmed in a multivariate model including “most ESA responsive” and “ESA hyporesponsive” patients. However, as no association between serum phosphate and hemoglobin was tested in this study, this effect could be due to the FGF23 level. 

In our analysis, a negative association between ESA and hemoglobin was detected. This could be explained by the fact that, due to the retrospective real-life design of our study, ESA was prescribed only in patients with lower hemoglobin, according to current Clinical Practice Guidelines [40]. For this reason, it can be considered as confounding by indication. 

Conversely to the phosphate impact, we found that the assumption of phosphate binder seemed to lightly improve the hemoglobin concentration. This is in line with other studies both in rats [41] and in humans [42,43], which reported an increase in hemoglobin in patients with phosphorous binder. This association, however, could be due to confounding by indication, because phosphate binders are taken by patients with hyperphosphatemia, in turn related to higher hemoglobin. However, even when performing the analysis and excluding phosphate binders from the multivariate model, the results did not differ.

Our study has strengths and limitations. The main strength is the large sample size and the longitudinal design. To our knowledge, no other studies have directly evaluated the association between phosphate and hemoglobin in a large dialysis population. The limitations of our study include the observational design, which cannot exclude the presence of residual confounding, and the absence of information on FGF23 measurements and EPO dose, and also, the iron and ESA type were not detailed in our database. The lack of this information precludes the computation of the ESA hyporesponsiveness index. Furthermore, our analysis included only dialysis patients of Caucasian descent from a single Italian region, thus impairing the generalizability of our study.

## 5. Conclusions

In conclusion, in our study, performed in a unique large cohort of 6263 dialysis patients, we found that phosphate reduction linearly reduces hemoglobin concentration. This result seems to be independent of personal features, comorbidities, and drugs. In addition, even though our results are not conclusive, age could have an impact on this association. Further studies are needed to confirm this association and to detail if ESA doses can impact on it. Furthermore, the impact of phosphate, hemoglobin, and ESA doses on cardiovascular events and mortality deserve further studies in the future.

## Figures and Tables

**Table 1:** (**A**,**B**) Baseline characteristics of clinical, demographic, and laboratory data.

**A**	**Whole Sample** **(*n* = 6263)**
Hemoglobin (g/dL)	10.4 ± 1.3
Age (years)	66 ± 16
Sesso (male)	3904 (62.4%)
BMI (kg/m^2^)	25.8 ± 5.1
DBP (mmHg)	72 ± 13
SBP (mmHg)	137 ± 19
Arterial hypertension (yes/no)	2366 (37.8%)
COPD (yes/no)	289 (4.6%)
Chronic liver disease (yes/no)	214 (3.4%)
Diabetes mellitus (yes/no)	1079 (17.2%)
Hemiplegia (yes/no)	32 (0.5%)
Heart failure (yes/no)	293 (4.7%)
IBD (yes/no)	54 (0.9%)
Malignancies (yes/no)	285 (4.6%)
Metastasis (yes/no)	57 (0.9%)
Vascular disease (yes/no)	262 (4.2%)
Albumin (g/dL)	3.58 ± 0.56
Cholesterol (mg/dL)	158 ± 45
CRP (mg/dL)	2.9 [0.9–6.6]
Ferritin (ng/mL)	148 [16–314]
Serum glucose (mg/dL)	119 ± 48
HCO_3_ (mmol/L)	21.8 ± 3.1
KT/V	1.32 ± 0.33
iPTH (pg/mL)	228 [112–391]
Potassium (mmol/L)	4.9 ± 0.8
Triglycerides (mg/dL)	140 ± 79
Transferrin saturation (%)	23 ± 15
**B**	**Whole Sample** **(*n* = 6263)**
ACEI (yes/no)	460 (7.3%)
Folic acid (yes/no)	586 (9.4%)
Calcium carbonate (yes/no)	514 (8.2%)
Cholecalciferol (yes/no)	138 (2.2%)
Cortisone therapy (yes/no)	166 (2.7%)
Dementia (yes/no)	80 (1.3%)
Diuretic (yes/no)	1627 (26%)
ESA (yes/no)	2900 (46.3%)
Iron supplementation (yes/no)	0 (0%)
Immunosuppression (yes/no)	15 (0.2%)
PPI (yes/no)	2373 (37.9%)
Paricalcitol (yes/no)	486 (7.8%)
Sevelamer (yes/no)	1469 (23.5%)
Vitamin B12 (yes/no)	176 (2.8%)

BMI: body mass index; COPD: chronic obstructive pulmonary disease; IBD: inflammatory Bowel Diseases; DBP: diastolic blood pressure; iPTH: intact parathormone; CRP: C-reactive protein; SBP: systolic blood pressure. ACEI: angiotensin convertase enzyme inhibitor; ESA: erythropoietin-stimulating agent; PPI: protonic pump inhibitors.

## Data Availability

The data were retrieved from the Sicilian Registry of Nephrology, Dialysis, and Transplantation (http://www.crtsicilia.it/PUBLIC/RegistroRSNDT/CentriDialisiETx.aspx (accessed on 6 July 2022)).

## Supplementary Materials

The following supporting information can be downloaded at: https://www.mdpi.com/article/10.3390/jcm13195657/s1, Table S1: Baseline correlated of serum phosphate and hemoglobin.; Table S2: Linear Mixed Model showing the direct association between serum phosphate and hemoglobin.; Table S3: Hosmann sensitivity analysis in LMM; Dependent variable: hemoglobin; Figure S1. Diagram for inclusion process.
