# Supplementary material for: Impact of Serum Phosphate on Hemoglobin Level: A Longitudinal Analysis on a Large Cohort of Dialysis Patients
Source: J Clin Med. 2024 Sep 24;13(19):5657. doi: 10.3390/jcm13195657 (PMC11477030; doi:10.3390/jcm13195657)
Supplement: Supplementary file 1 [file jcm-13-05657-s001.zip › jcm-3214299-supplementary.pdf]

SUPPLEMENTARY

Table S1. Baseline correlated of serum phosphate and hemoglobin.

	**Serum phosphate (mg/dl)**		**Hemoglobin (g/dl)**	
	r	**p**	R	**p**
Serum phosphate (mg/dL)	1		**0.042**	**0.001**
Hemoglobin (g/dL)	**0.042**	**0.001**	**1**	
AceI (yes/no)	0.024	0.060	-0.002	0.890
Folic acid (yes/no)	**-0.044**	**<0.001**	-0.013	0.286
Albumin (g/dL)	**0.128**	**<0.001**	**0.230**	**<0.001**
BMI (kg/m^2)	**0.054**	**<0.001**	-0.012	0.399
Calcium carbonate (yes/no)	-0.02	0.108	-0.008	0.540
Cholecalciferol (yes/no)	-0.01	0.490	0.01	0.430
Cortisone treatment (yes/no)	0.022	0.084	-0.003	0.836
Dementia (yes/no)	**-0.044**	**0.001**	**-0.033**	**0.009**
Diabetes (yes/no)	**-0.055**	**<0.001**	**-0.056**	**<0.001**
Diuretics (yes/no)	-0.006	0.650	**-0.035**	**<0.001**
Hemiplegia (yes/no)	-0.02	0.113	**-0.027**	**0.035**
Liver disease (yes/No)	0.001	0.957	**0.044**	**0.001**
ESA (yes/no)	-0.007	**0.565**	**-0.195**	**<0.001**
Age (year)	**-0.252**	**<0.001**	-0.015	0.247
Serum ferritin (ng/mL)	-0.016	0.21	-0.050	0.004
Iron supplementation(yes/no)	**-0.02**	**0.050**	-0.015	**<0.001**
Bicarbonate (mmol/L)	**-0.119**	**<0.001**	**0.043**	**0.053**
Immunosuppression (yes/no)	**0.026**	**0.037**	**-0.018**	**0.149**
PPI (yes/no)	**-0.037**	**0.003**	0	1
Arterial hypertension (yes/no)	-0.002	0.852	**-0.019**	**0.125**
KT/V	**-0.037**	**0.016**	**0.124**	**<0.001**
COPD (yes/no)	**-0.027**	**0.034**	**-0.058**	**<0.001**
Vascular disease (yes/no)	**-0.024**	**0.056**	-0.014	0.259
IBD (yes/no)	-0.023	0.075	-0.011	0.389
Malignancies (yes/no)	**-0.031**	**0.013**	**-0.034**	**0.007**
DBP (mmHg)	**0.134**	**<0.001**	0.015	0.314
Paracalcitolo (yes/no)	0.012	0.331	**0.019**	**0.139**

SBP (mmHg)	**0.080**	**<0.001**	**-0.022**	**0.131**
Phosphate binder (yes/no)	**0.086**	**<0.001**	**0.067**	**<0.001**
Potassium (mmol/L)	**0.177**	**<0.001**	**0.102**	**<0.001**
CRP (mg/dL)	**-0.099**	**<0.001**	**-0.170**	**<0.001**
iPTH (pg/mL)	**0.185**	**<0.001**	-0.005	0.743
Heart failure (yes/no)	**-0.029**	**0.024**	**-0.043**	**0.001**
Sevelamer (yes/no)	**0.096**	**<0.001**	**0.085**	**<0.001**
Transferrin saturation (%)	0.002	0.477	0.017	**<0.001**
Metastasis (yes/no)	**-0.025**	**0.046**	**-0.030**	**0.017**
Vitamin B12 (yes/no)	**-0.032**	0.012	-0.008	0.548

AceI: angiotensin convertase enzyme inhibitor; BMI: Body mass index; COPD: chronic obstructive pulmonary disease; DBP: Diastolic blood pressure; ESA: erythropoietin stimulating agent; IBD: inflammatory bowel diseases; iPTH: intact parathormone; PPI: protonic pump inhibitors; CRP= C-Reactive Protein; SBP: Systolic blood pressure.

Table S2 Linear Mixed Model showing the direct association between serum phosphate and hemoglobin.

	Univariate model			Multivariate model		
	β	95% CI	p	β	95% CI	p
Serum phosphate [ln (mg/dl)]	0.39	0.36/0.41	<0.001	0.14	0.04/0.24	0.006
Sex				0.15	0.07/0.23	0.001
Serum potassium (mmol/l)				0.12	0.08/0.16	<0.001
Calcium carbonate (yes/no)				-0.07	-0.17/0.02	0.15
Folic acid (yes/no)				0.07	-0.02/0.16	0.11
Iron supplementation (yes/no)				0.08	0.03/0.14	0.002
ESA (yes/no)				-0.81	-0.87/-0.75	<0.001
Paracalcitol (yes/no)				0.03	-0.04/0.10	0.37
Immunosuppressors (yes/no)				0.88	-0.81/2.58	0.31
Calcitriol (yes/no)				0.05	-0.01/0.12	0.10
Heart failure (yes/no)				-0.10	-0.24/0.03	0.12
Periferal vascular disease (yes/no)				-0.001	-0.11/0.11	0.99
Diabetes (yes/no)				0.04	-0.05/0.13	0.41
Chronic liver disease (yes/no)				0.12	-0.02/0.26	0.10
Visit				0.003	-0.001/0.006	0.11
KT/V				0.14	0.06/0.23	<0.001

BMI (kg/m^2)		-0.005	-0.01/0.002	0.19
iPTH (pg/ml)		-0.80e-5	-0.0001/0.0001	0.87
Transferrin saturation (%)		0.01	0.01/0.01	<0.001
Serum ferritin (ng/ml)		-0.0001	-0.0002/-0.00004	0.01
CRP (mg/dl)		-0.003	-0.005/-0.001	0.01
Serum bicarbonate (mmol/l)		-0.02	-0.03/-0.01	<0.001
Serum calcium (mg/dl)		0.09	0.06/0.12	<0.001
SBP (mmHg)		-0.003	-0.004/-0.001	<0.001
Age (year)		0.002	-0.001/0.005	0.20
Phosphate binders (yes/no)		0.15	0.08/0.22	<0.001
AceI (yes/no)		0.04	-0.09/0.10	0.94
PPI (yes/no)		0.15	0.08/0.23	<0.001
Arterial hypertension (yes/no)		-0.02	-0.09/0.04	0.52
Vitamin B12 (yes/no)		-0.05	-0.17/0.06	0.37
Diuretic (yes/no)		0.05	-0.04/0.14	0.27
Cinacalcet (yes/no)		0.04	-0.05/0.12	0.37
Dementia (yes/no)		-0.02	-0.27/0.23	0.90
Malignancies w/o metastasis (yes/no)		0.03	-0.08/0.14	0.55
COPD (yes/no)		0.10	-0.02/0.23	0.09

AceI: angiotensin convertase enzyme inhibitor; BMI: Body mass index; COPD: chronic obstructive pulmonary disease; DBP: Diastolic blood pressure; ESA: erythropoietin stimulating agent; IBD: inflammatory bowel diseases; iPTH: intact parathormone; PPI: protonic pump inhibitors; CRP: C-Reactive Protein; SBP: Systolic blood pressure.

Table S3 Hosmann sensitivity analysis in LMM. Dependent variable: hemoglobin.

	β	95% CI	p
Serum phosphate [ln(mg/dl)]	0.16	0.06/0.26	0.001
Sex	0.15	0.06/0.23	0.001
Iron supplementation (yes/no)	0.08	0.03/0.14	0.001
ESA (yes/no)	-0.81	-0.87/-0.76	<0.001
Visit	0.003	0.001/0.01	0.02
KT/V	0.16	0.08/0.24	<0.001
Transferrin saturation (%)	0.01	0.01/0.01	<0.001
C Reactive Protein (mg/dl)	-0.004	-0.006/-0.001	0.001
Serum bicarbonate (mmol/l)	-0.02	-0.03/-0.02	<0.001

Serum calcium (mg/dl)	0.09	0.06/0.12	<0.001
SBP (mmHg)	-0.002	-0.003/-0.001	0.001
PPI (yes/no)	0.18	0.11/0.25	<0.001
Phosphate binder (yes/no)	0.18	0.11/0.24	<0.001

ESA: erythropoietin stimulating agent; IBD: inflammatory bowel diseases; PPI: protonic pump inhibitors; SBP: Systolic blood pressure.

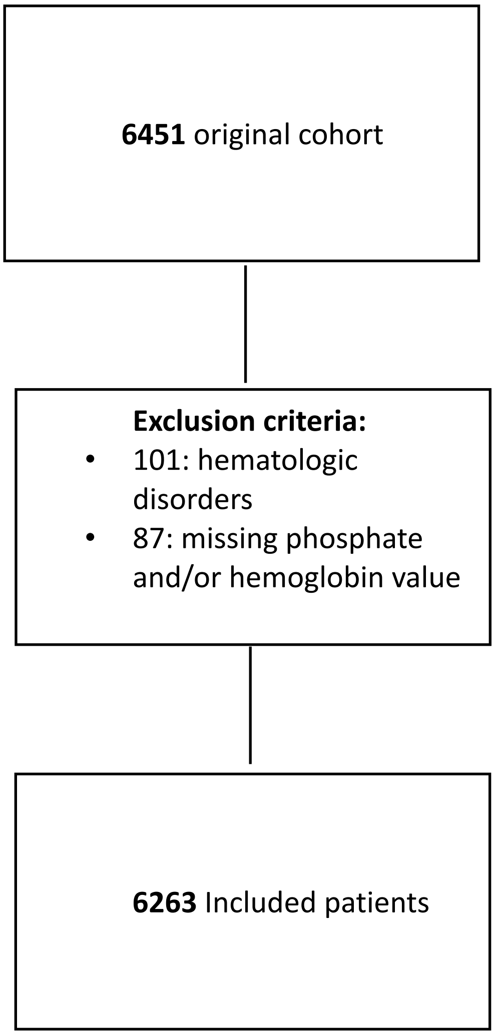

Figure S1. Diagram for inclusion process.
